# Enhancement of Fungal Enzyme Production by Radio-Frequency Electromagnetic Fields

**DOI:** 10.3390/jof8111187

**Published:** 2022-11-10

**Authors:** Mayura Veerana, Nan-Nan Yu, Si-Jin Bae, Ikhwan Kim, Eun-Seong Kim, Wirinthip Ketya, Hak-Yong Lee, Nam-Young Kim, Gyungsoon Park

**Affiliations:** 1 Radio-Frequency Integrated Circuit (RFIC) Center, Kwangwoon University, Seoul 01897, Korea; 2 Plasma Bioscience Research Center, Department of Plasma-Bio Display, Kwangwoon University, Seoul 01897, Korea; 3 Department of Electronics Engineering, Kwangwoon University, Seoul 01897, Korea; 4 Department of Electrical and Biological Physics, Kwangwoon University, Seoul 01897, Korea

**Keywords:** radiofrequency electromagnetic field, α-amylase, *Aspergillus oryzae*, fungus, intracellular calcium

## Abstract

Enzyme production by microorganisms on an industrial scale has demonstrated technical bottlenecks, such as low efficiency in enzyme expression and extracellular secretion. In this study, as a potential tool for overcoming these technical limits, radio-frequency electromagnetic field (RF-EMF) exposure was examined for its possibility to enhance production of an enzyme, α-amylase, in a filamentous fungus, *Aspergillus oryzae*. The RF-EMF perfectly resonated at 2 GHz with directivity radiation pattern and peak gain of 0.5 dB (0.01 Watt). Total protein concentration and activity of α-amylase measured in media were about 1.5–3-fold higher in the RF-EMF exposed (10 min) sample than control (no RF-EMF) during incubation (the highest increase after 16 h). The level of α-amylase mRNA in cells was approximately 2–8-fold increased 16 and 24 h after RF-EMF exposure for 10 min. An increase in vesicle accumulation within fungal hyphae and the transcription of some genes involved in protein cellular trafficking was observed in RF-EMF-exposed samples. Membrane potential was not changed, but the intracellular Ca^2+^ level was elevated after RF-EMF exposure. Our results suggest that RF-EMF can increase the extracellular level of fungal total proteins and α-amylase activity and the intracellular level of Ca^2+^.

## 1. Introduction

Enzymes of microbial origin have demonstrated their usefulness in the food industry, medicine, agriculture, bioremediation, and many other industries [1]. Enzymes produced by microorganisms have several advantages compared to those of animal and plant origin, such as higher stability under various physical and chemical conditions and faster microbial growth [1,2,3]. Because microorganisms can propagate much faster in smaller volumes than animals and plants and conditions for culture are not complicated, a sufficient number of microbes for industrial-scale production of enzymes can quickly be obtained, and therefore, enzyme production can be manipulated easily with economic efficiency [2].

Since the Nagoya protocol on access to genetic resources has been effective, microorganisms and genetic resources producing useful enzymes have become valuable economic resources to nations [4]. Therefore, the quest for new enzyme-producing microbial strains and the improvement of efficiency in enzyme production by microorganisms appears to be more important than before. Because finding new microbial species or strains takes a great deal of time, the qualitative improvement of enzyme production processes by existent microorganisms may be more practical and efficient. To improve the efficiency of mass enzyme production by microorganisms, some technical barriers should be overcome. One is the low yield of enzyme protein produced in a microbial cell although microorganisms are growing faster than plants and animals. Studies have often shown that both native (host gene) and heterologous (non-host gene) enzymes are produced with low efficiency [5,6,7,8]. The other is inefficiency in cellular trafficking of secretory vesicles containing enzymes for extracellular secretion [9]. These two barriers can play a critical role in determining the success of mass enzyme production of which the process consists of several steps such as a search for the proper microbial species and strains, development of an enzyme expression system and engineered strains, mass culture of microorganisms, and isolation and purification of enzymes [10,11]. Particularly, enzymes secreted into media are much easier to isolate and purify than those inside cells, and thus, enhancement of secretion is a way to reduce costs for isolation and purification step in the process. An improvement in the capability of microorganisms to secrete enzymes extracellularly is important as well as expression of intracellular enzyme genes.

Technical development for the improvement of microbial enzyme production has occasionally been reported, and the approach using genetic engineering is most frequently explored [12,13]. Genetically engineered microbial strains have contributed to promoting the efficiency of enzyme production, but the safety issues on genetically modified organisms such as negative influence on ecosystem and human health and gene transfer have restricted the application range. Recently, we demonstrated that treatment of fungal spores with non-thermal atmospheric pressure plasma could promote the secretion of fungal enzymes into media [14]. Physical tools may be able to reduce genetic risks and be considered as a potential solution for the improvement of enzyme production. As another physical tool, RF-EMF was examined for the enhancement of fungal enzyme production in this study. Heat generated by RF-EMF has frequently been applied to inactivate microorganisms [15,16,17,18,19,20]. In addition, studies have shown that RF-EMF can accelerate wound healing by the regulation of keratinocyte proliferation and migration, expression of genes involved in wound healing, and inflammation [21,22]. Although RF-EMF has demonstrated enhancing effects on wound healing in a mammalian system, it has hardly been explored in the functional activation of microorganisms. In this study, we aimed to examine the possibility of RF-EMF to promote enzyme production by microorganisms. This investigation is essential for expanding the knowledge spectrum of activation effects of RF-EMF in cells and organisms, which have not been actively explored. The effects of RF-EMF on intracellular production and extracellular secretion of an enzyme, α-amylase, were analyzed in a filamentous fermenting fungus, *Aspergillus oryzae*.

*A. oryzae* is a beneficial fungus that is actively used in the food fermentation industry because it can produce various extracellular enzymes with high industrial value such as α-amylase, protease, pectinase, and β-galactosidase [23,24]. Because fungus is composed of eukaryotic cells, our investigation will be useful to enlarge the spectrum of knowledge about the effects of RF-EMF on microbial or eukaryotic cell. *A. oryzae* strain KACC47488 used as a model organism in our study is an industrial strain from a fermentation company in Korea, and its biology has been well characterized [25]. This strain is a good producer of α-amylase, an industrially useful enzyme [23,24], and therefore, evaluating the effects of RF-EMF on this fungal strain can provide useful information applicable to developing technologies for industrial-scale enzyme production.

## 2. Materials and Methods

### 2.1. Fungal Strain and Growth Conditions

The Korean Agricultural Culture Collection (KACC) at the National Institute of Agricultural Sciences, Rural Development Administration (Wanju-gun, Jeollabuk-do, Korea) provided a fungal strain, *Aspergillus oryzae* KACC47488, which was used in this study. Potato dextrose agar (PDA) and potato dextrose broth (PDB) (MB cell, Los Angeles, CA, USA) were used as media for propagating and culturing the fungus, and the fungus was cultured at 30 °C in the dark.

### 2.2. Exposure of Fungal Spores to Radio-Frequency Electromagnetic Fields (RF-EMF)

A microstrip patch antenna was constructed for generating radiofrequency electromagnetic fields (RF-EMF). Microstrip patch antenna is well known for low-profile, compact size, and directivity radiation beam pattern. Because of these features, the microstrip patch antenna has been widely used in wireless communication system [26,27,28]. Recently, it is applied to various biomedical sensors [29,30].

The microstrip patch antenna was designed by following Equations (1)–(3) [31]. In Equations (1)–(3), W and L are width and length of the patch radiator; c is the speed of light, which is $3\times 10^{8}$ m/s; $f_{r}$ is the target resonant frequency of the antenna; $\varepsilon_{r}$ is the relative permittivity of the substrate (dielectric); $\varepsilon_{0}$ and $\mu_{0}$ are the permittivity and permeability of free space, respectively; $\varepsilon_{reff}$ is the effective relative permittivity which occurs by the fringe effect; and h is the height of the substrate. Equation (3) can be calculated when the width of the patch is larger than the height of the substrate.

(1) $$W=\frac{c}{2f_{r}}\sqrt{\frac{2}{\varepsilon_{r}+1}}$$

(2) $$L=\frac{1}{2f_{r}\sqrt{\varepsilon_{reff}}\sqrt{\mu_{0}\varepsilon_{0}}}-2\Delta L$$

(3) $$\varepsilon_{reff}=\frac{\varepsilon_{r}+1}{2}+\frac{\varepsilon_{r}-1}{2}{\left[1+12\frac{h}{W}\right]}^{-1}$$

The direct coaxial feeding method was used to match the microstrip patch antenna impedance. The position of the RF connector (P) was calculated by using Equation (4) to achieve impedance matching of the microstrip patch antenna [32]. In Equation (4), *Z_A_*(*P*) is the impedance when the RF connector is connected at (P). For matching impedance 50 Ω, we can assume $Z_{A}\left(P\right)$ is 50. $Z_{A\;edge}$ is the edge impedance of the microstrip patch radiator. L is the length of the patch radiator.

(4) $$Z_{A}\left(P\right)=Z_{A\;edge}\;cos^{2}\left(\frac{\pi P}{L}\right)$$

**Figure 1:**
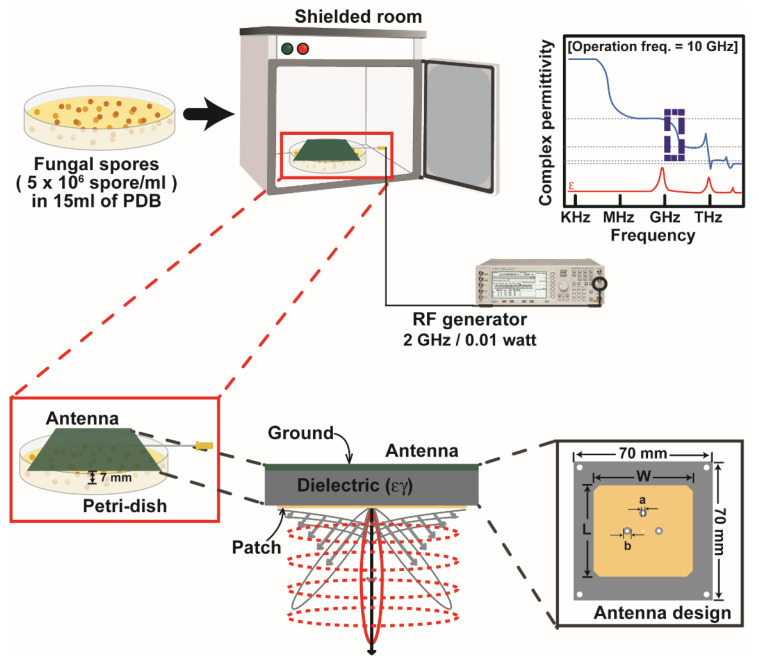
Schematic view of the structure of the antenna and RF (radio-frequency) generator producing RF-EMF (radio-frequency electromagnetic field) and exposure of fungal spores in PDB (potato dextrose broth) medium.

Figure 1 shows the schematic view of the proposed microstrip patch antenna for generating RF-EMF. The microstrip patch antenna for RF-EMF was constructed using a Teflon substrate (*h* = 1 mm, $\varepsilon_{r}=2.1,$ tan δ = 0.001) and Ansys HFSS 21. The proposed microstrip patch antenna’s target resonant frequency was 2 GHz. The total dimensions of the antenna were 70 (w) × 70 (d) × 1 (h) mm with 40 × 39.5 mm microstrip radiator.

The reflection coefficient, peak gain, and normalized radiation pattern of the proposed microstrip patch antenna for RF-EMF were analyzed (Supplementary Figure S1). For this analysis, the microstrip patch antenna was excited by a vector signal generator (E4438C, Agilent Technologies, Santa Clara, CA, USA). The proposed antenna perfectly resonated at 2 GHz with a directivity radiation pattern and peak gain of 0.5 dB (Supplementary Figure S1).

For exposure to RF-EMF, *A. oryzae* spores were collected from culture plates that had been cultured for one week. The plates were filled with 15 mL sterile phosphate-buffered saline (PBS) and then the fungal mycelia were scraped using a spreader. Four layers of sterile miracloth (Calbiochem, Darmstadt, Germany) were used to filter the fungal suspension. The filtered fungal suspension was centrifuged at 3134× *g* for 5 min, and the supernatant was removed. The spore pellet was resuspended in new PDB solution, making the concentration 5 × 10^6^ spores per mL. The fungal spore suspension (15 mL) was placed in a Petri dish (90 mm in diameter), and the microstrip patch antenna (RF-EMF) or plastic cover (no RF-EMF; control) was put onto the Petri dish, as shown in Figure 1. The distance between the surface of the fungal spore suspension and antenna was 7 mm. Fungal spores were then subjected to RF-EMF generated from the antenna (2 GHz, 0.01 W) for the indicated time (Figure 1). At the same time, control Petri dish was placed in a shielded container without RF-EMF generation for the indicated time. After exposure, the spore suspension exposed to RF-EMF or none (control) was transferred to an Erlenmeyer flask (100 mL) and incubated at 30 °C and shaken for the indicated time.

### 2.3. Measurement of the Total Protein Level and α-Amylase Activity in the Media

The spore suspension (15 mL, 5 × 10^6^/mL) was exposed to RF-EMF and then transferred to a 100 mL Erlenmeyer flask. The flask was incubated at 30 °C and shaken for the indicated time (16, 24, and 48 h). Culture media were collected at each time point, and total protein concentration was measured using a Bradford protein assay kit (Bio-Rad, Hercules, CA, USA) following the manufacturer’s protocol.

The α-amylase activity in media was measured as described previously [14]. The reaction mixture containing 100 µL of diluted cultured media and 100 µL of 1% (*w*/*v*) soluble starch in 0.1 M acetate buffer (pH 5.6) was incubated at 50 °C for 30 min, and the 3,5-dinitrosalicylic acid assay was used to determine the liberated reducing sugars (product; maltose equivalent). The cultured media with water instead of soluble starch was used as a blank to eliminate the non-enzymatic release of sugars. A standard curve was created using maltose. One unit (U) of amylase activity was defined as the amount of enzyme needed for producing 1 μg of maltose (as reducing sugar equivalent) per mL per minute under the assay conditions. Specific activity was expressed as amylase activity (U) per mg of protein.

### 2.4. Analysis of Extracellular α-Amylase Levels Using Aptamers

The level of α-amylase protein in media was qualitatively analyzed using an α-amylase-specific aptamer labeled with fluorescence. The sequence of *A. oryzae* α-amylase aptamer used in this study (Table 1) was designed following that described in a previous study [33].

Following RF-EMF exposure, the spore suspension was incubated at 30 °C and shaken for 16 h. The cultured media was collected, and 5 μL of media was mixed with or without 3 μL of 10 μM α-amylase aptamer labeled with fluorescence. The mixture was incubated at 25 °C in the dark for 3 h. The commercially available solution of *A. oryzae* α-amylase (1 mg/mL) (Sigma-Aldrich, St. Louis, MO, USA) was used as a positive control. After incubation, the mixture solution was applied to native polyacrylamide gel (8% in 1 × TBE) electrophoresis. Electrophoresis was processed at output voltage 80 V for 40 min using a Mini-PROTEAN^®^ Tetra Vertical Electrophoresis System (BioRad, Hercules, CA, USA). After electrophoresis, the gel was examined and photographed using the ChemiDocTM MP Imaging System (BioRad, Hercules, CA, USA). The α-amylase band was detected at 532 nm and the intensity of the band was analyzed using the Image Lab Touch Software version 3.0.1 (BioRad, Hercules, CA, USA).

### 2.5. Membrane Potential Assay and Vesicle Staining

To analyze fungal membrane potential and vesicles, spores suspended in PDB (15 mL, 5 × 10^6^ spores/mL) were exposed to RF-EMF for 10 min. The unexposed sample was used as a control. The spore suspension was then incubated at 30 °C and shaken for 16 h after exposure, and the fungal hyphae were washed in sterile PBS. For membrane potential, the washed fungal hyphae were incubated in 1 mL of PBS containing 50 μg of bis (1,3-dibutylbarbituric acid) trimethine oxonol (DiBAC4(3), Invitrogen, Carlsbad, CA, USA) for 1 h at 4 °C in the dark. After incubation, fungal hyphae were washed using PBS, and the fluorescence was examined using a confocal laser scanning microscope at 490 (excitation) and 516 (emission) nm (FV-1000 MPE spectra, Olympus Corporation, Tokyo, Japan).

For vesicle staining, the washed fungal hyphae were stained with FM4-64 (N-(3-Triethylammoniumpropyl)-4-(6-(4-(Diethylamino) Phenyl) Hexatrienyl) Pyridinium Dibromide; Invitrogen) prepared in PBS (25 μM) at room temperature for 30 min in the dark. Fluorescence intensity was analyzed at 514 (excitation) and 670 (emission) nm using the confocal laser scanning microscope (Olympus Corporation).

### 2.6. Detection of Intracellular Calcium Ions

Intracellular calcium ions (Ca^2+^) in fungal hyphae were detected using a fluorescent calcium indicator, Fluo-3 AM (N-(3-Triethylammoniumpropyl)-4-(6-(4-(Diethylamino) Phenyl) Hexatrienyl) Pyridinium Dibromide; Invitrogen, Carlsbad, CA, USA). Fungal spores suspended in PDB (15 mL, 5 × 10^6^ spores/mL) were exposed to RF-EMF for 10 min and then incubated at 30 °C and shaken for 16 h. The unexposed sample was used as a control. After washing in sterile PBS, fungal hyphae were incubated in 10 μM Fluo-3 in PBS at 30 °C for 30 min and washed with PBS, and fluorescence was detected at 506 (excitation) and 526 (emission) nm using a confocal laser scanning microscope (FV-1000 MPE spectra, Olympus Corporation, Tokyo, Japan).

### 2.7. Quantitative PCR Analysis

To measure the mRNA expression levels of target genes, fungal spores in PDB media (15 mL, 5 × 10^6^ fungal spores/mL) were exposed to RF-EMF for 10 min and cultured at 30 °C and shaken for 16 and 24 h. The fungal hyphae were harvested and washed with deionized (DI) water twice and stored at −80 °C until total RNA extraction. TaKaRa RNAiso Plus kit (TaKaRa Bio, Tokyo, Japan) was used to extract total RNA according to the manufacturer’s instructions, and the NanoDrop (Biotek Instruments, VT, USA) was used to measure the concentration of RNA. The equal amount of RNA (100 ng) was taken to synthesize cDNA, and cDNA was synthesized using a synthesis kit, the ReverTra Ace qPCR RT Master Mix with gDNA Remover (Toyobo, Osaka, Japan), following the manufacturer’s instructions. cDNA of each target gene was amplified and quantified at every thermal cycle using iQ SYBR Green Supermix (Biorad) and a real time RT-PCR thermocycler (CFX96TM, Biorad), as previously described [34]. Primer sequences of target genes are listed in Table 2. The level of mRNA for each target gene was determined as that normalized to a reference gene (18S ribosomal RNA); mRNA level of target gene = 2^-∆∆Ct^, where ∆∆Ct = (Ct _target_—Ct _reference_) _RF exposure_—(Ct _target_—Ct _reference_) _control_ [35].

### 2.8. Measurement of pH and Levels of Hydrogen Peroxide (H_2_O_2_), Nitrogen Oxides (NOx) and Inositol 1,4,5-Triphosphates (IP_3_)

The pH of treated media was measured using a portable pH meter (Oakton Instruments, Vernon Hills, IL, USA). To measure H_2_O_2_ and NOx levels in background media, 15 mL PDB was exposed to RF-EMF for 10 min. Unexposed media were used as the control. Following exposure, PDB media were incubated at 30 °C and shaken for 0, 16, 24, and 48 h. Concentrations of H_2_O_2_ and NOx were then measured using Amplex™ Red Hydrogen Peroxide/Peroxidase Assay Kit (Molecular Probes, Eugene, OR, USA) and QuantiChrom^TM^ Nitric Oxide Assay Kit (BioAssay Systems, Hayward, CA, USA), respectively, following the manufacturer’s protocols.

To assess the intracellular level of inositol 1,4,5-triphosphates (IP_3_), fungal mycelia were harvested at 24 h after exposure to RF-EMF and immediately ground with liquid nitrogen. Deionized water was added into the ground powder and mixed. The mixture was centrifuged at 5000× *g* for 5 min, and then the supernatant was collected and stored at −80 °C until analysis. The IP_3_ concentration was determined using the Inositol Triphos-phate Elisa Kit (BioVision, Waltham, MA, USA) following the manufacturer’s protocol. Briefly, 50 μL of the supernatant sample and 50 μL of the biotinylated detection antibody working solution were placed and mixed in each well of a 96-well microplate according to the instructions. The plate was incubated at 37 °C for 45 min, and then 100 μL of HRP (Horse Radish Peroxidase) conjugate working solution was added and incubated at 37 °C for 30 min. After washing five times with wash buffer, 90 μL of substrate reagent was added into each well and the plate was incubated at 37 °C for 15 min. Finally, the reaction was stopped with 50 μL of stop solution and detected immediately at 450 nm with a microplate reader (Biotek, Winooski, VT, USA).

### 2.9. Statistical Analysis

Student’s *t*-test was used to statistically analyze the data, and statistical significance was indicated as follows: * denotes *p* < 0.05, ** denotes *p* < 0.01, and *** denotes *p* < 0.001.

## 3. Results

### 3.1. Production of α-Amylase was Enhanced in the Fungus after Exposure to RF-EMF

We exposed the fungal spores in PDB to RF-EMF generated at 2 GHz under different electrical power and exposure time conditions. The level of α-amylase activity in the media was generally higher when 0.01 W was used than when 0.05 W was used for all incubations (Supplementary Figure S2). No major difference was observed between exposure times of 10 and 30 min (Supplementary Figure S2). Therefore, we used RF-EMF generated under 2 GHz and 0.01 W in further experiments.

**Figure 2:**
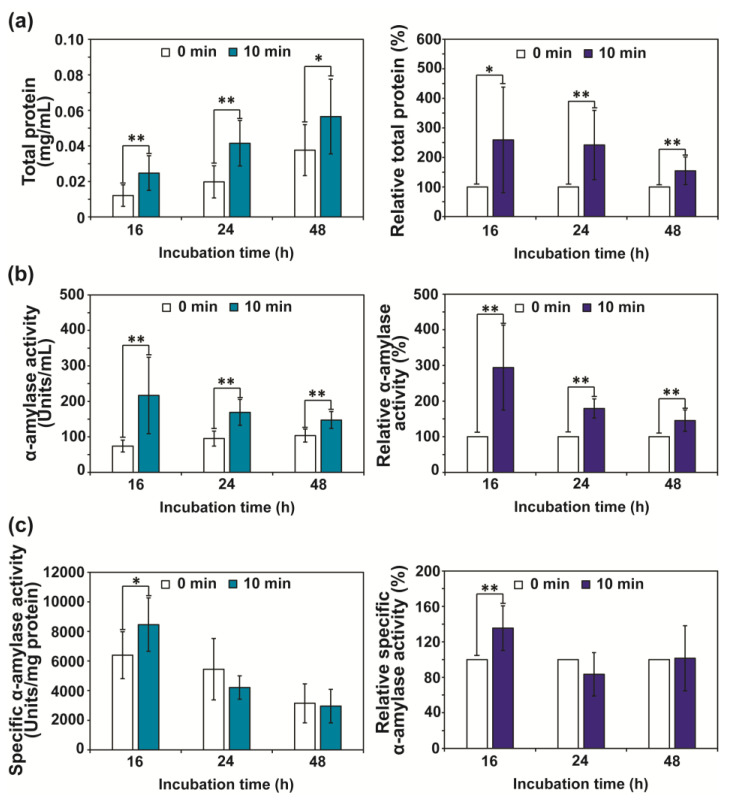
Level of total proteins and activity of α-amylase in media (PDB) at 16, 24, and 48 h after fungal spores in PDB were unexposed (control) and exposed to RF-EMF (2 GHz, 0.01 W) for 10 min. Each value represents the mean of nine replicate measurements. The experiment was repeated three times, each with three replicate measurements: * *p* < 0.05 and ** *p* < 0.01: (**a**) Amounts of total proteins in media; (**b**) Activity of α-amylase; (**c**) Specific activity of α-amylase.

After exposure to RF-EMF, the concentration of total protein in the media was significantly increased (Figure 2a). Approximately 1.5–2.5 times higher total protein compared to that of the control (no RF-EMF exposure) was detected after 16, 24, and 48 h, and the rate of increase was reduced with longer incubation duration (Figure 2a). The level of α-amylase activity in the media as an indicator of extracellularly secreted α-amylase was increased at 16, 24, and 48 h after RF-EMF exposure, showing a 300%, 180%, and 150% increase in enzyme activity, respectively (Figure 2b). The specific activity of α-amylase was significantly higher in media with RF-EMF treated fungal hyphae, compared to media of the unexposed control, after 16 h (Figure 2c). However, no significant difference in specific activity was observed between plasma treated and control samples after 24 and 48 h (Figure 2c).

We tried to detect the level of α-amylase protein in media using *A. oryzae* α-amylase-specific aptamer. The difference in band intensity (indicating α-amylase protein level) was very subtle between control and RF-treated sample, and bands in positive and negative controls were not properly shown (Supplementary Figure S3). To examine if RF-EMF can alter the protein structure of secreted α-amylase and enhance the level of enzyme activity, we applied RF-EMF directly to α-amylase dissolved in VM media (2 U/mL) and then measured α-amylase activity. α-Amylase activity measured after 16 and 24 h was not significantly different between the control (no RF-EMF) and RF-EMF-exposed samples (Supplementary Figure S4).

**Figure 3:**
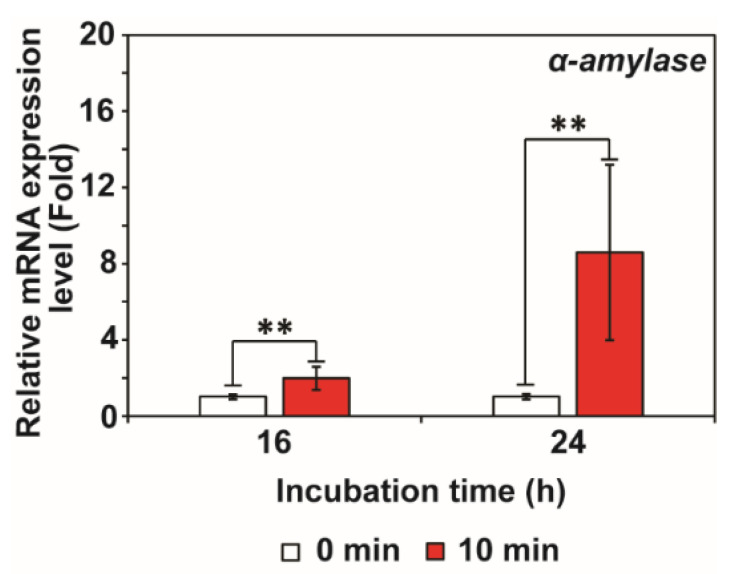
Transcription of α-amylase in fungal hyphae after exposure to RF-EMF. Level of mRNA in fungal cells was measured at 16 and 24 h after fungal spores were unexposed (control) and exposed to RF-EMF (2 GHz, 0.01 W) for 10 min. Each value represents the mean of nine replicate measurements. The experiment was repeated three times, each with three replicate measurements: ** *p* < 0.01.

The level of α-amylase mRNA was significantly increased in fungal hyphae exposed to RF-EMF after 16 and 24 h (Figure 3). Approximately two- and eight-fold greater amounts of α-amylase mRNA were measured in RF-EMF exposed fungal hyphae compared to the control (unexposed fungal hyphae) after 16 and 24 h, respectively (Figure 3).

### 3.2. RF-EMF Enhanced Vesicle Accumulation and the Transcription of Genes Involved in Cellular Trafficking

**Figure 4:**
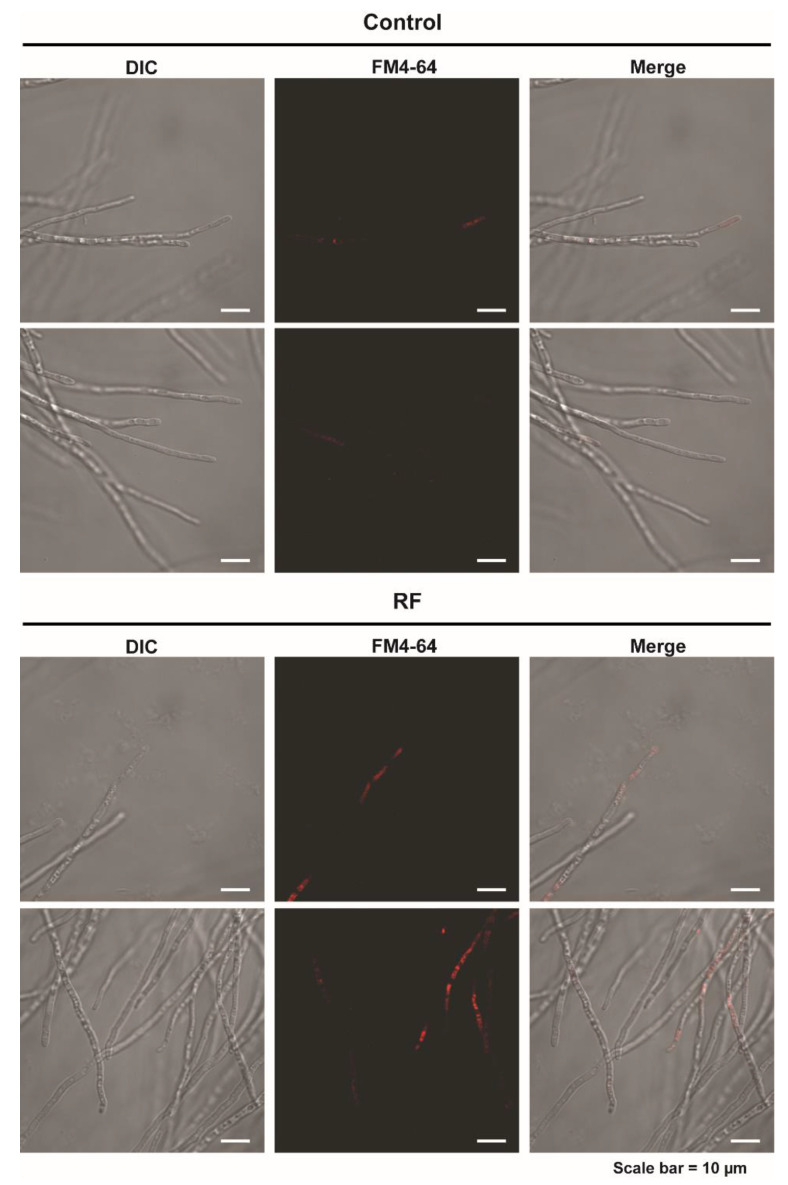
Staining of vesicles using FM4-64 in fungal hyphae at 16 h after fungal spores were un- exposed (control) and exposed to RF-EMF (2 GHz, 0.01 W) for 10 min.

The increased level of α-amylase activity in media after RF-EMF exposure indicates that RF-EMF can possibly activate the enzyme secretory pathway. To test this hypothesis, accumulation of secretory vesicles in fungal hyphae and expression of genes involved in trafficking of secretory vesicles were examined. After staining vesicles with FM4-64, a fluorescent dye, fungal hyphae of the RF-EMF-exposed sample showed fluorescence more frequently than the control hyphae (no RF-EMF exposure) (Figure 4 and Supplementary Figure S5). This indicates the elevated accumulation of vesicles in the RF-EMF exposed fungal hyphae than control. In RF-EMF-exposed samples, fluorescence was observed in apical and subapical areas of fungal hyphae (Figure 4 and Supplementary Figure S5).

**Figure 5:**
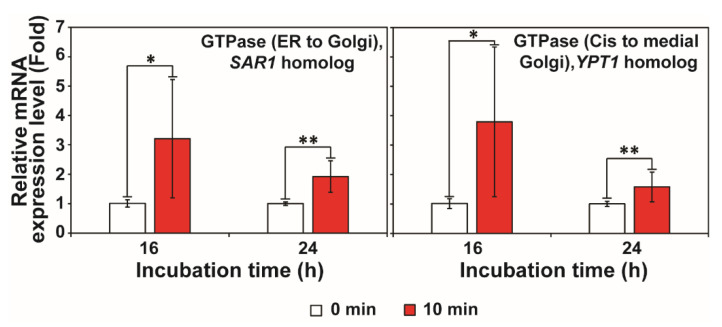
Transcription level of genes involved in regulating cellular trafficking in *A. oryzae* after exposure to RF-EMF (2 GHz, 0.01 W). Each value represents the mean of nine replicate measurements. The experiment was repeated three times, each with three replicate measurements: * *p* < 0.05 and ** *p* < 0.01.

We also observed that transcription of some genes involved in regulating cellular trafficking of secretory proteins was significantly increased after exposure to RF-EMF (Figure 5).

### 3.3. RF-EMF Exposure Enhanced the Level of Intracellular Ca^2+^ in Fungal Hyphae

**Figure 6:**
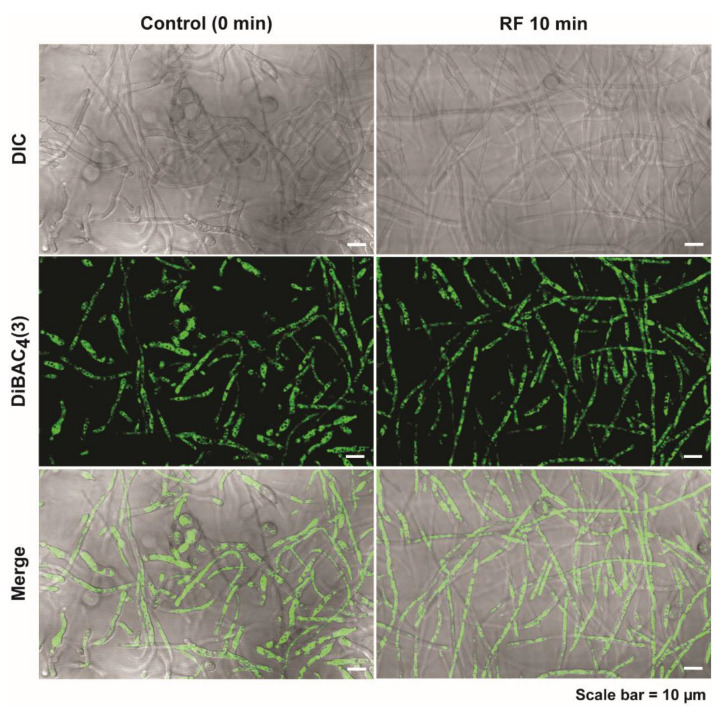
Membrane potential assay using a fluorescent indicator, DiBAC4(3), in fungal hyphae at 16 h after fungal spores were unexposed (control) and exposed to RF-EMF (2 GHz, 0.01 W) for 10 min.

In our previous study, we found that the increase in intracellular vesicle level was associated with membrane depolarization and elevation of intracellular Ca^2+^ level [14]. Similarly, we also examined the membrane potential and Ca^2+^ level in fungal hyphae after RF-EMF exposure in this study. Fungal hyphal membrane potential assessed using a fluorescent indicator, DiBAC4(3), was not significantly changed after exposure to RF-EMF, indicating no further membrane depolarization by RF-EMF (Figure 6 and Supplementary Figure S6).

**Figure 7:**
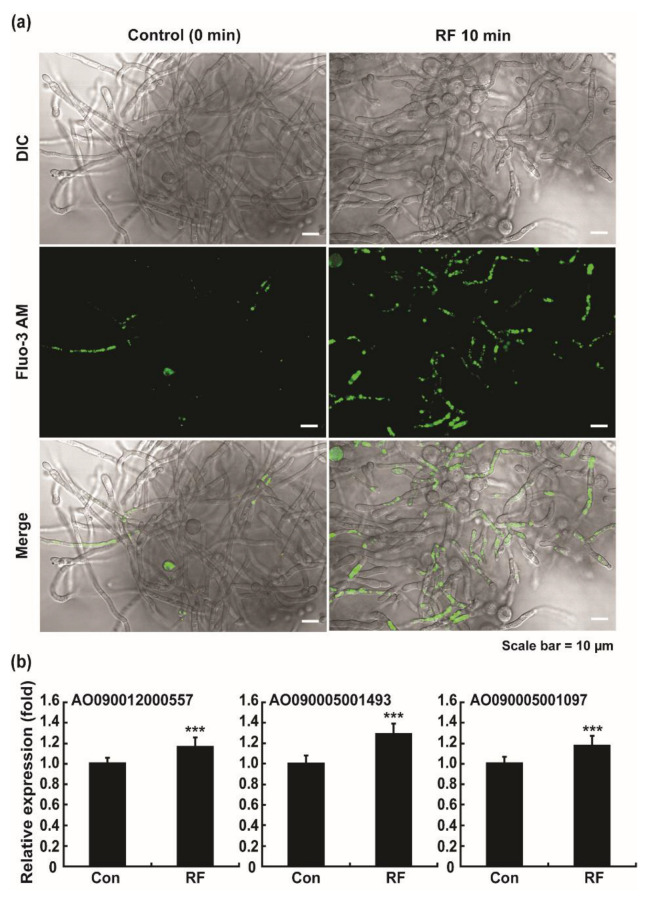
Assay for intracellular Ca^2+^ using Fluo-3 AM (**a**) and level of transcription of three phospholipase C (PLC) genes (**b**) in fungal hyphae at 16 h after no exposure (control) or RF-EMF exposure (2 GHz, 0.01 W) for 10 min. In (**b**), each value represents the mean of nine replicate measurements. The experiment was repeated three times, each with three replicate measurements: *** *p* < 0.001.

We stained fungal hyphae using a fluorescent Ca^2+^ indicator and found a greater area of fluorescence in RF-EMF exposed fungal hyphae than in the control (no RF-EMF exposure), indicating that the intracellular Ca^2+^ level was increased after RF-EMF exposure (Figure 7a and Supplementary Figure S7). Intracellular Ca^2+^ is known as the secondary signal, and its level is controlled by Ca^2+^ pumps and channels on the membrane and phospholipase C (PLC) [36]. Because the intracellular Ca^2+^ level was elevated upon RF-EMF exposure, we examined the expression of phospholipase C (PLC) in *A. oryzae* hyphae after RF-EMF exposure. Transcription level of three PLC genes in *A. oryzae* (AO090012000557, AO090005001593, and AO090005001097) was significantly increased in fungal hyphae after RF-EMF exposure (Figure 7b). Membrane PLC is known to hydrolyze PIP_2_ (phosphatidylinositol-4,5-bisphosphate) producing IP_3_ (inositol1,4,5-triphosphate), and IP_3_ induces the elevation of intracellular Ca^2+^ levels [34]. We measured the level of IP_3_ in fungal hyphae after exposure to RF-EMF. However, no significant difference in intracellular IP_3_ level was found between fungal hyphae with and without RF-EMF exposure (Supplementary Figure S8).

### 3.4. Analysis for the Level of H_2_O_2_, NOx, and pH

**Figure 8:**
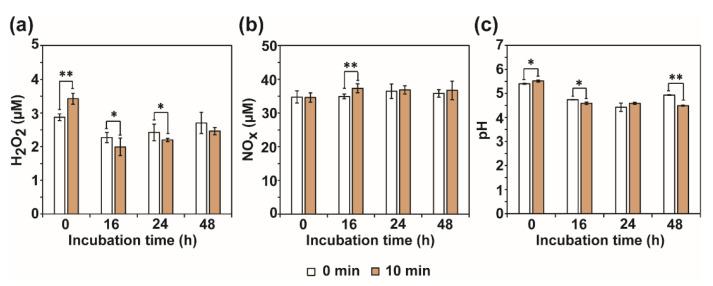
Levels of H_2_O_2_ (**a**), Nox (**b**), and pH (**c**) in media after exposure to RF-EMF (2 GHz, 0.01 W). Each value represents the mean of nine replicate measurements. The experiment was repeated three times, each with three replicate measurements: * *p* < 0.05 and ** *p* < 0.01.

To find the RF-EMF factors that affected enzyme production, levels of H_2_O_2_, NOx, and pH in media after RF-EMF exposure were examined. Levels of H_2_O_2_ were significantly increased in PDB medium immediately after exposure to RF-EMF (Figure 8a and Supplementary Table S1). NOx concentration was significantly increased after 16 h of RF-EMF exposure (Figure 8b and Supplementary Table S2). However, concentrations of both H_2_O_2_ and NOx were not significantly different between the control and RF-EMF exposure with longer incubation duration (Figure 8a,b and Supplementary Tables S1 and S2). Media pH was slightly elevated immediately after RF-EMF exposure (0 h), but decreased after 16 and 48 h (Figure 8c and Supplementary Table S3).

## 4. Discussion

Radio-frequency (RF) waves have often been explored in microbial inactivation and pasteurization of foods [15,16]. Studies show that microorganisms are inactivated by the thermal effect generated by RF waves [17,18,19,20]. However, recent studies suggest that the non-thermal effect of RF can play an important role in microbial inactivation [19,37]. An accumulating number of studies have demonstrated various effects of RF-EMF on microbial cells. RF-EMF emitted from mobile phones and Wi-Fi routers is observed to alter the antibiotic susceptibility and growth of a pathogenic bacteria (the increased antibiotic susceptibility after longer exposure) [38]. Genes involved in glucose transportation and tricarboxylic acid (TCA) cycle were highly expressed in a budding yeast under RF-EMF exposure [39]. In contrast to the previous studies (mostly focused on inactivation of microorganisms), our study demonstrates a potential role of RF-EMF that can promote the functional cellular processes in microorganisms. Studies have shown that RF electromagnetic fields and waves can improve wound healing and increase the production or activation of cytokines and proteins involved in wound healing in mammalian systems [21,22,40,41]. However, reports on the activation of beneficial microorganisms are scarce. To our knowledge, our study provides the first demonstration showing that RF-EMF (electromagnetic field) can activate the extracellular enzyme (α-amylase) production in a beneficial microorganism (fungus), probably through enhancing the intracellular enzyme expression and the extracellular enzyme activity.

Activation of cellular processes during wound healing often occurs under low radio-frequency electromagnetic waves [21,40]. In our study, a high frequency (2 GHz) and low power (0.01 W) were used. The thermal effect may not be critical in our study because heat was not produced in media (fungal spores were submerged in media) after RF-EMF exposure. Although the frequency level was high, the low electrical power used in generating RF-EMF may have played an important role in enhancing enzyme production in our study. Whether the frequency level or electric power is important for activation of cellular processes is not clear. This will be further investigated in the future.

The efficiency of RF-EMF observed in our study may not be comparable or superior to that of fungal strain improvement by genetic modification in promoting enzyme production. The improved α-amylase production was previously observed in genetically modified *A. oryzae* generated by random mutagenesis [42]. Efficiency rate of α-amylase production in *A. oryzae* mutant strain was higher than that of RF-EMF observed in our study. In addition, the enhancement effect of RF-EMF was the highest after 16 h and then weakened after 48 and 72 h, probably because of reduction in freshness of the culture condition over time. Microbial strain improvement by genetic manipulation has been attempted to increase the enzyme production efficiency in other studies [12,13]. Although efficiency of RF-EMF is not as high as the genetically modified *A. oryzae* strain, RF-EMF is relatively advantageous to safety issues compared to genetically modified microorganisms and can be applied as additional use together with genetically modified microorganisms.

Our results demonstrate that RF-EMF may be able to enhance protein secretion because total protein level detected in media and level of vesicles (secretory) in fungal hyphae are significantly higher in the RF-EMF-treated sample than control. In addition, α-amylase activity in media was significantly elevated after RF-EMF treatment. High variation in values of total protein concentration and α-amylase activity may be caused by heterogeneity of data among three repeated experiments. It is not clear whether the enhanced α-amylase activity in media is a result of increased secretion of α-amylase protein into media or not, because our preliminary analysis using α-amylase specific aptamer shows no clear difference in enzyme protein level between the control and RF-EMF-treated sample (Supplementary Figure S3). More analysis using measurement tools may be needed. RF-EMF does not seem to specifically enhance the secretion of α-amylase because the change in levels of total protein and α-amylase activity in media showed similar pattern over incubation time.

RF-EMF may possibly be involved in regulating the intracellular expression of proteins. As a consequence, the extracellular level of total proteins and α-amylase activity may be increased. We observed a significant increase in intracellular mRNA level of α-amylase in the RF-EMF-treated sample. However, these mRNA levels were measured at 16 and 24 h after RF-EMF treatment, which was in the same time range as the elevated extracellular activity of α-amylase was observed. Therefore, our data only showed that transcription of α-amylase in the RF-EMF-treated sample was maintained at an elevated level until enzyme secretion. Further analysis on gene expression during time earlier than protein secretion will be needed to clarify the relationship.

Another point is that increase in protein secretion and α-amylase activity in media after RF-EMF treatment can be possibly resulted from the promotion of fungal growth. If RF-EMF has accelerated the fungal hyphal growth, more enzymes can be obtained from the increased mass of fungal mycelia. We observed no visual difference in fungal mycelial growth between control and RF-EMF-treated sample. This indicates that RF-EMF can enhance extracellular enzyme production by other mechanism(s) rather than by promoting fungal growth. However, quantitative analysis on the effect of RF-EMF on fungal growth should be further investigated. In addition, swollen round structures in fungal hyphae shown in Figure 7a may not be malformed structures generated after RF-EMF treatment. Those may be the swollen and aggregated spores that are still present during early stage of germination (16 h). Therefore, these swollen round structures may not be involved in the regulation of enzyme production.

From our analysis, we observed several cellular and molecular responses of fungal cells to RF-EMF, and these observations could provide clues for elucidating the mechanism(s) of RF-EMF action on fungal enzyme production. First, RF-EMF does not change *A. oryzae* cell membrane potential. No change in membrane potential was observed after exposure to RF-EMF in previous studies [43,44,45]. Interestingly, we observed that non-thermal atmospheric pressure plasma changed the membrane potential of *A. oryzae* in our previous study [14]. This may be because RF-EMF and plasma have different ionizing or reactivity properties. Although RF-EMF can penetrate deeper into the cell and tissue than plasma, RF-EMF does not have ionizing ability, while plasma generating various reactive species has high oxidizing ability and reactivity. Therefore, it is possible that plasma affects membrane electric properties more strongly than RF-EMF, and this results in the different outcomes in membrane potential change between RF-EMF and plasma.

Our results also show that RF-EMF can elevate the level of Ca^2+^ inside the fungal cell. This observation accords with the previous discoveries in which Ca^2+^ influx or efflux was regulated in response to RF-EMF in various cells (for a review, see [45]). It is not clear whether intracellular Ca^2+^ is released from intracellular membranous organelles or through Ca^2+^ channels in plasma membrane. mRNA levels of three phospholipase C (PLC) genes in *A. oryzae* hyphae were increased after RF-EMF treatment. PLC is known to mediate the cleavage of PIP_2_ (phosphatidylinositol (PI)-4,5-bisphosphate) in membranes into IP_3_ (inositol 1,4,5 triphosphate), which induces increasing intracellular Ca^2+^ levels [36,46]. We found no significant change in the level of IP_3_ in fungal hyphae after RF exposure (Supplementary Figure S7). Although RF-EMF seems to cause the elevation of Ca^2+^ level in fungal hyphae, involvement of PLC and further Ca^2+^ signaling needs to be further examined to elucidate the relationship between RF-EMF and Ca^2+^ generation in fungal cells.

Increased levels of Ca^2+^ in *A. oryzae* hyphae may possibly regulate the promoted transcription of GTPase genes involved in cellular trafficking as shown in our data. An increase in the expression of cellular trafficking genes is likely to activate protein secretion. We observed that vesicle accumulation was enhanced in RF-EMF exposed fungal hyphae, indicating that the elevation of enzyme secretion might be through the activation of cellular vesicle trafficking. Ca^2+^ is known as a secondary messenger in the cell and leads to the expression of genes involved in fungal growth and differentiation [36]. Regulation of protein cellular trafficking by Ca^2+^ is often observed in mammalian cells [47] but not frequently in fungal cells. Calcium containing phosphopeptides are known to improve protein secretion and cellular trafficking in fungi [48]. It is also possible that the increase in Ca^2+^ levels in fungal hyphae observed in our study can enhance Ca^2+^ association with proteins that regulate cellular trafficking and secretion.

Although RF-EMF exposure caused the increase in intracellular Ca^2+^ levels and gene expression, how RF-EMF is involved in regulating these cellular processes is still unclear. Several hypotheses can be suggested. First, RF-EMF may be able to stimulate the cellular molecules in fungal hyphae, which eventually lead to elevation of Ca^2+^ and gene transcription in cells. RF waves can penetrate cells and affect the activation of cellular molecules [44]. RF energy is not strong enough to ionize molecules but cause vibration or polarization of molecules by changing the electron distribution in the atom [49]. Polarization and vibration of intracellular molecules may possibly be involved in activating molecules and downstream cellular processes, such as elevation of the Ca^2+^ level and gene expression. Although we do not have evidence of molecules polarized or vibrated by RF-EMF, it may be a topic for further investigation. Secondly, there may possibly be a magnetosensor or receptor molecule to electromagnetic fields of RF in fungi. Cryptochromes are known as magnetosensors responding to static magnetic fields in many organisms including fungi [50]. Photolyase involved in DNA repair is a well-known cryptochrome in fungi [51]. Sensing magnetic fields has been found as another function of crytochrome in Drosophila, plants, and birds [52,53]. In *Arabidopsis thaliana*, cryptochrome is found as a receptor of the RF electromagnetic field [53]. A cryptochrome in *Aspergillus nidulans* regulates sexual differentiation [54]. If there is a cryptochrome sensing RF-EMF in *A. oryzae*, activated cryptochrome by RF-EMF can be a possible mediator explaining the missing link between RF-EMF and intracellular Ca^2+^ elevation in our study.

## 5. Conclusions

Our study demonstrates a potential role for RF-EMF in enhancing the extracellular level of total protein and α-amylase activity, in a filamentous fungus, *A. oryzae*. An intracellular molecular change in response to RF-EMF exposure is the elevation of the Ca^2+^ level. Ca^2+^ may have acted as a secondary signal that leads to the activation of pathways involved in intracellular gene expression and extracellular secretion of α-amylase. Future investigations may be focused on determining the link between RF-EMF exposure and Ca^2+^ elevation and how increased levels of Ca^2+^ regulate gene expression and enzyme secretion. Enzymes of microbial origin have assumed considerable industrial and economic importance, and therefore, overcoming technical limitations in mass enzyme production is essential for the prosperity of the enzyme industry. Our results suggest that RF-EMF can be a promising tool to promote extracellular enzyme production.

## Figures and Tables

**Table 1:** Sequence of an α-amylase specific aptamer (75 mers).

Primer Name	Primer Sequences
α-amylase aptamer *	5′-GGATACCTTAACGCCGCCTATTGTGAACGACGTGAATCGTGTTTGTGGGTCCGGAGTTGCACCCGTCTCGAAATC-3′

* Sequence is presented in the 5′ to 3′ direction. This aptamer has the same nucleotide sequence (no chemical modification) as that in a previous study [33].

**Table 2:** List of primers used in qPCR.

Genes	Primer Sequences
α-Amylase	Forward-5′ACTGGGTGGGATCATTGGTA3′ Reverse-5′ACAAGTGTAGGCCGGATCAC3′
GTPase (ER to Golgi), SAR1 homolog	Forward-5′CGAAGTGAGCGGTATCGTTT3′ Reverse-5′CCCTTTCCTGTGGTCTGGTA3′
GTPase (cis to medial Golgi), YPT1 homolog	Forward-5′TGATGGCAAGACAGTGAAGC3′ Reverse-5′TTGACACCCTCAGTGGCATA3′
AO090012000557	Forward-5′CCAGTCTGCCTCCTCTGTCTAGC3′ Reverse-5′TGGTTGTGAACGAGCGTCTATCTTG3′
AO090005001593	Forward-5′TGGTGGCATTGAACTGGCAGAAC3′ Rverse-5′TCTAGCTGACGACGCACGATAGTAG3′
AO090005001097	Foward-5′ACAAAGGTGGAGACTGGAATGATGC3′ Rverse-5′TCGTGATTGAGGGATGCTTGGTTG3′
18S ribosomal RNA	Forward-5′GGAAACTCACCAGGTCCAGA3′ Reverse-5′AGCCGATAGTCCCCCTAAGA3′

## Data Availability

Not applicable.

## Supplementary Materials

The following supporting information can be downloaded at: https://www.mdpi.com/article/10.3390/jof8111187/s1, Figure S1: Reflection coefficient (a), peak gain (b), and normalized radiation pattern (c) of proposed microstrip patch antenna; Figure S2: Activity of α-amylase (a), amount of total protein (b), and specific activity of α-amylase (c) in media measured after RF-EMF exposure; Figure S3: Level of α-amylase protein in media analyzed using *A. oryzae* α-amylase specific aptamer 16 h after fungal spores in PDB were unexposed (control) and exposed to RF-EMF (2 GHz, 0.01 W) for 10 min; Figure S4: Effect of RF-EMF on α-amylase dissolved in PDB medium; Figure S5: Staining of vesicles in fungal hyphae using FM4-64; Figure S6: Assay for membrane potential of fungal hyphae; Figure S7: Staining intracellular Ca^2+^ in fungal hyphae; Figure S8: Level of IP_3_ in fungal hyphae; Table S1: Level of H_2_O_2_ in media; Table S2: Level of NOx in media; Table S3: pH level of media.
