# Supplementary material for: Enhancement of Fungal Enzyme Production by Radio-Frequency Electromagnetic Fields
Source: J Fungi (Basel). 2022 Nov 10;8(11):1187. doi: 10.3390/jof8111187 (PMC9695996; doi:10.3390/jof8111187)
Supplement: Supplementary file 1 [file jof-08-01187-s001.zip › Supplementary information.pdf]

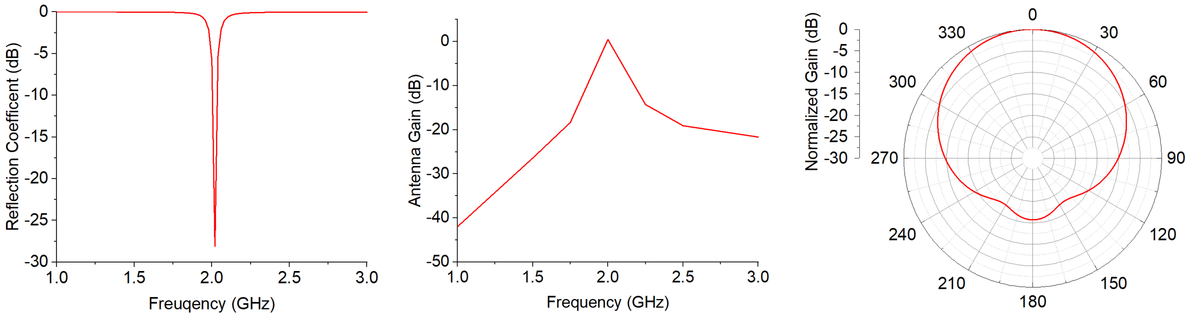

(a) **(b)** **(c)**

Supplementary Figure S1. Reflection coefficient (a), peak gain (b), and normalized radiation pattern (c) of proposed microstrip patch antenna.

(a)

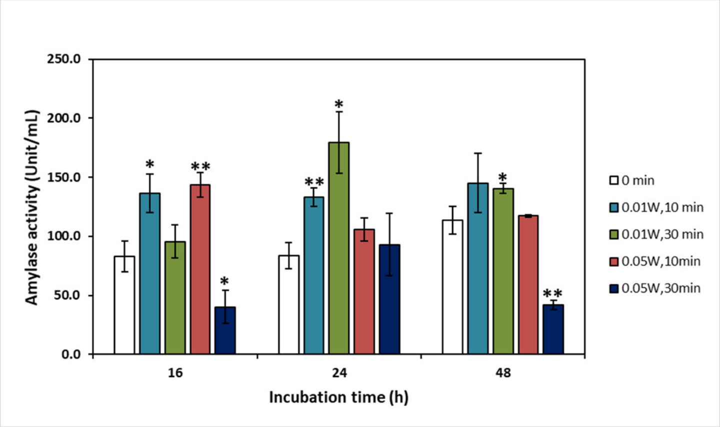

(b)

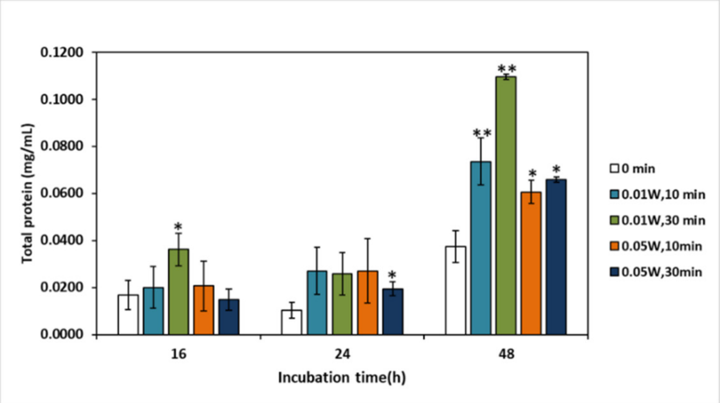

(c)

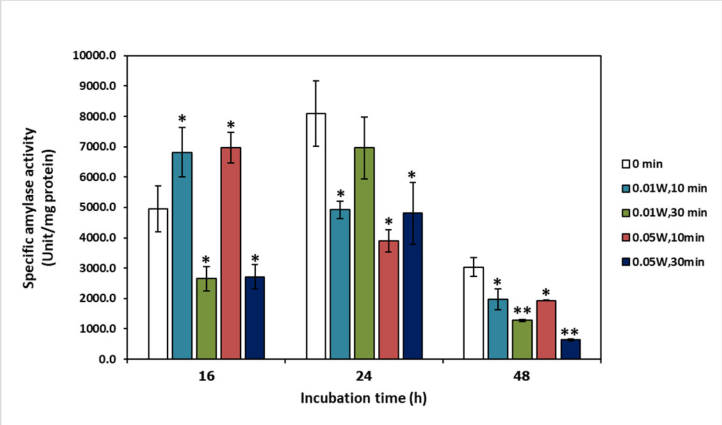

Supplementary Figure S2. Activity of α-amylase (a), amount of total protein (b), and specific activity of α-amylase (c) in media measured after RF-EMF (radio-frequency electromagnetic fields) exposure. Fungal spore suspension was exposed to RF-EMF (2 GHz, 0.01 or 0.05 W) for 0, 10 or 30 min. Each value represents the mean of 3 replicate measurements: *$p < 0.05$ and **$p < 0.01$.

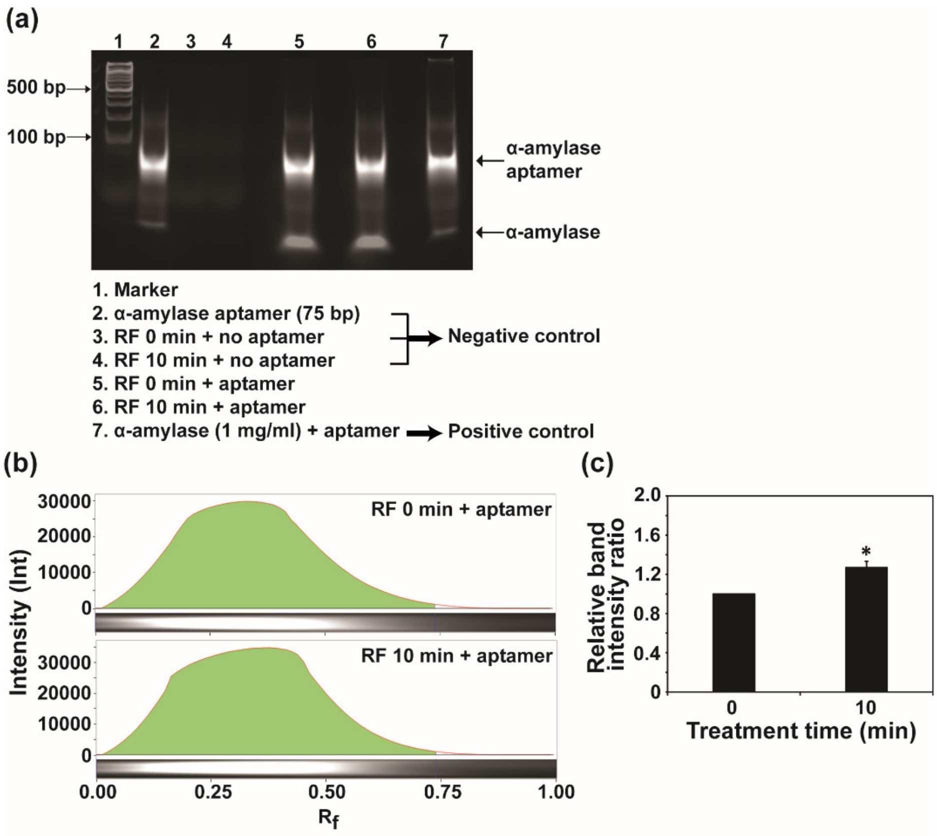

Supplementary Figure S3. Level of α-amylase protein in media analyzed using *A. oryzae* α-amylase specific aptamer 16 h after fungal spores in PDB were unexposed (control) and exposed to RF-EMF (2 GHz, 0.01 W) for 10 min: (a) α-amylase detected by specific aptamer on native polyacrylamide gel; (b) Intensity of α-amylase band estimated using Image Lab Touch Software version 3.0.1 (BioRad); (c) Ratio of α-amylase level between control and RF exposed samples.

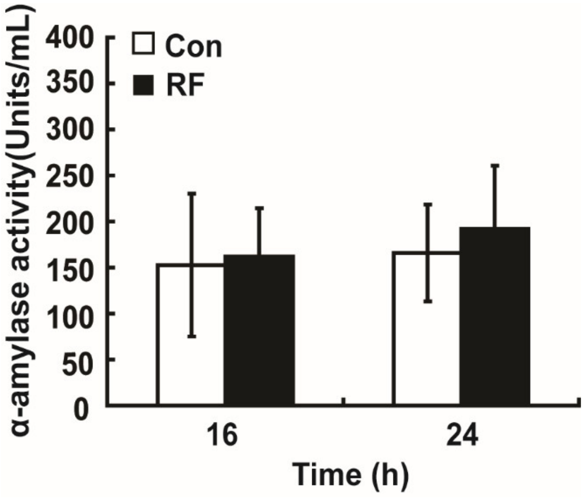

Supplementary Figure S4. Effect of RF-EMF on α-amylase dissolved in PDB medium (2 U/mL; 0.013 mg/mL). RF-EMF (2 GHz, 0.01 W) was applied to α-amylase solution for 10 min, and then samples were incubated for 16 and 24 h. Each value represents the mean of 4 replicate measurements.

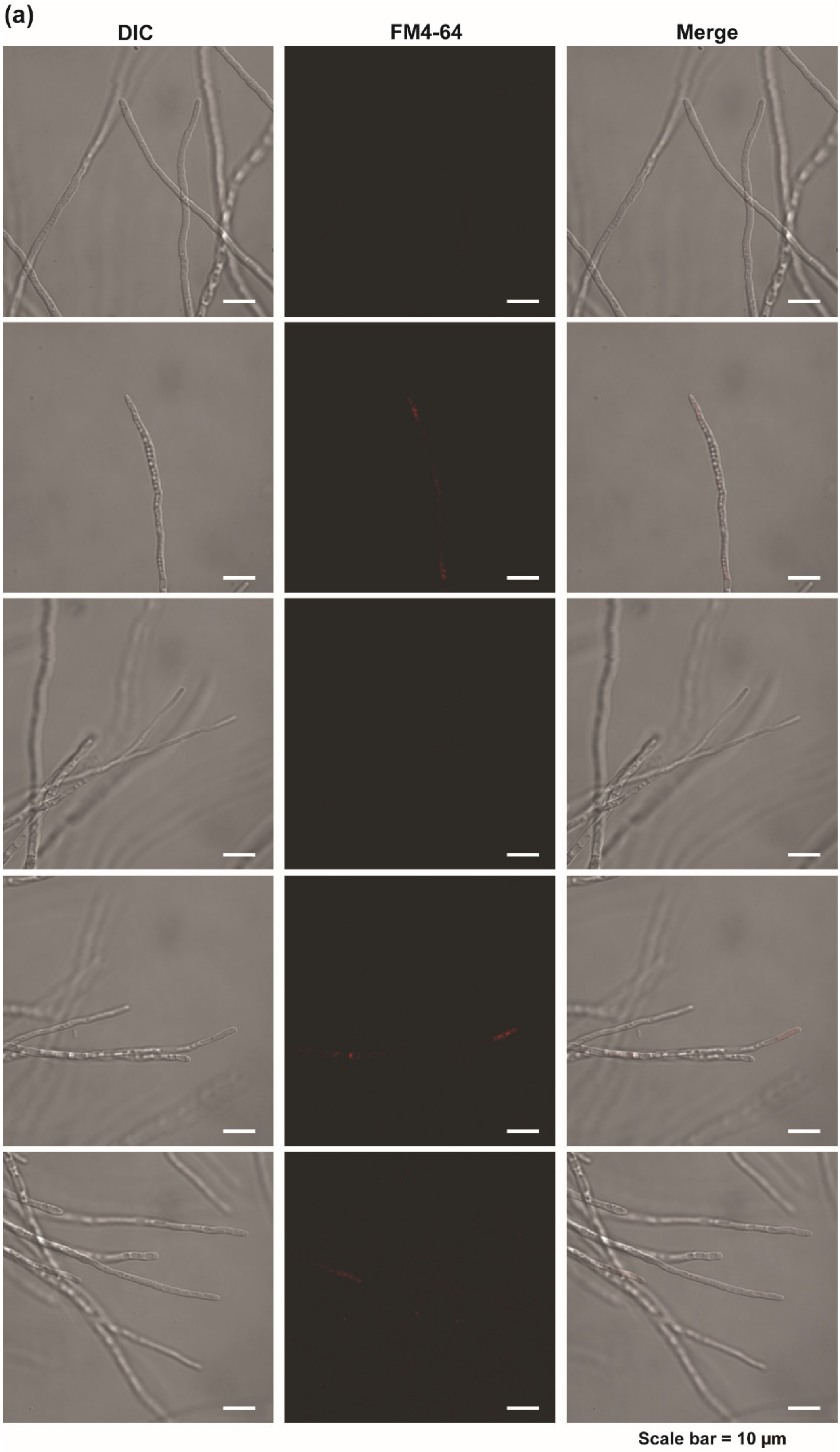
(a)
DIC
FM4-64
Merge
Scale bar = 10 μm

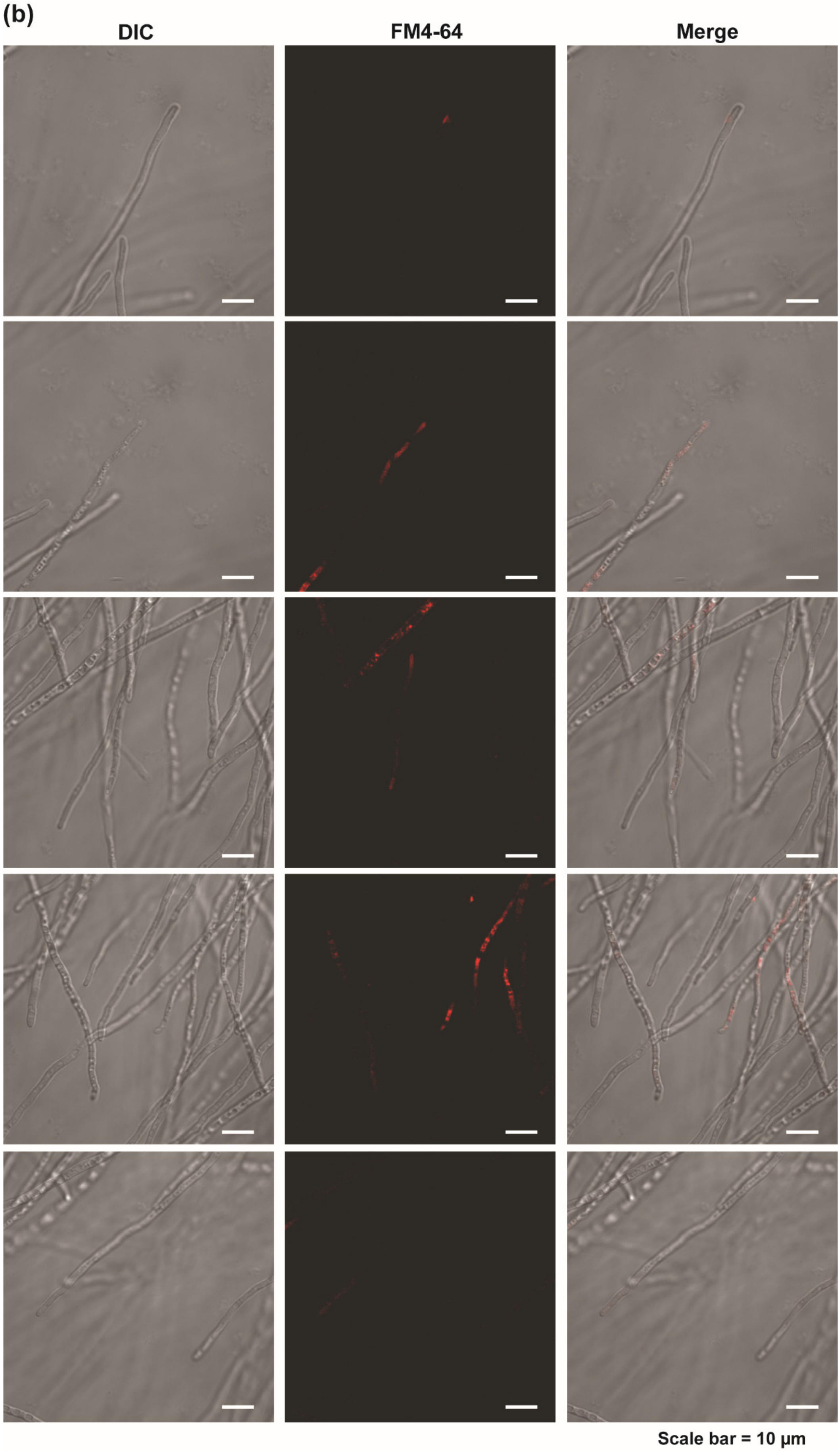
(b)
DIC
FM4-64
Merge
Scale bar = 10 μm

Supplementary Figure S5. Staining of vesicles in fungal hyphae using FM4-64. Fungal hyphae were stained at 16 h after spores were exposed to none (control) (a) and RF-EMF (2 GHz, 0.01 W) for 10 min (b).

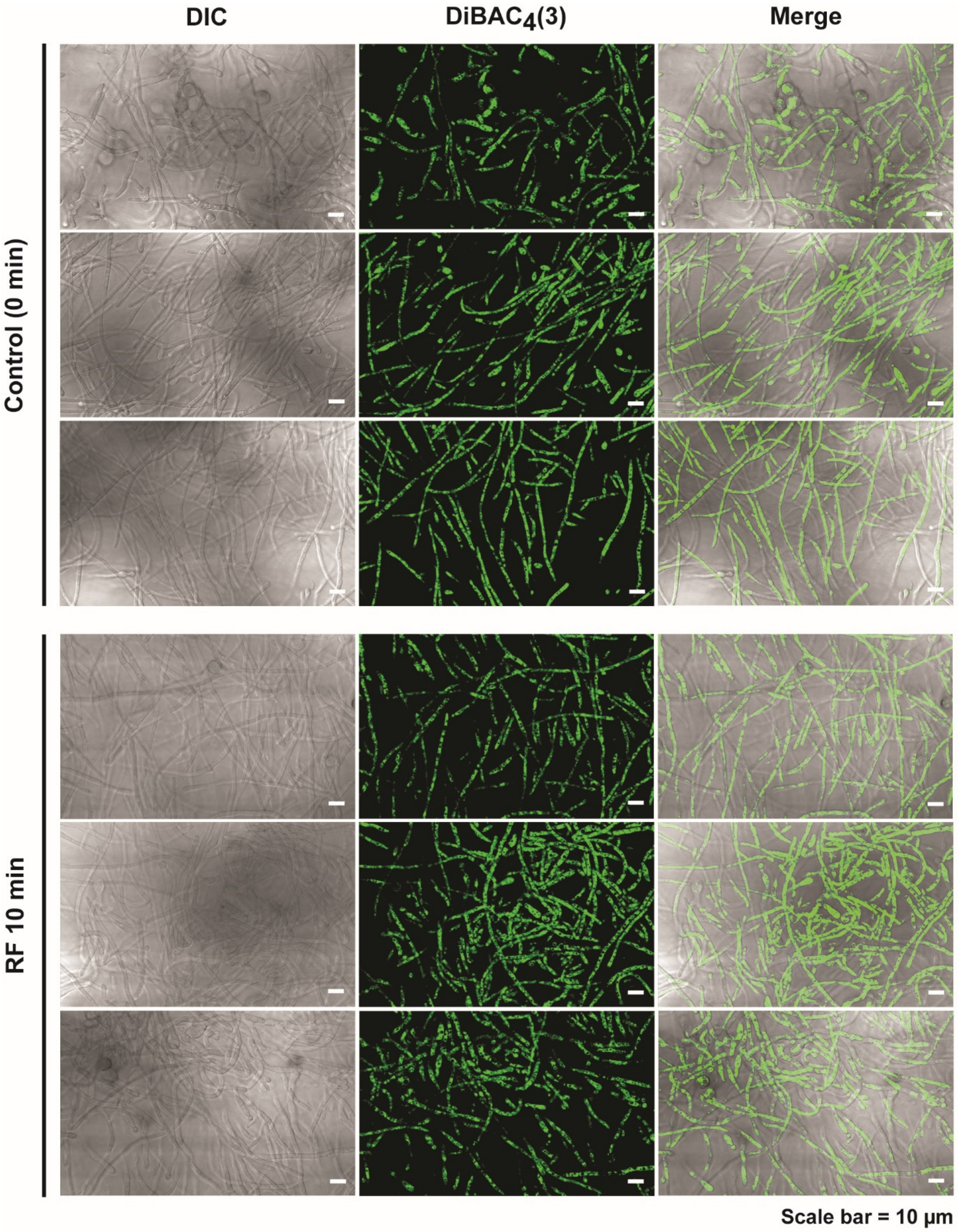

Supplementary Figure S6. Assay for membrane potential of fungal hyphae. A fluorescent indicator DiBAC$_4$(3) was applied to fungal hyphae at 16 h after spores were exposed to none (control) and RF-EMF (2 GHz, 0.01 W) for 10 min.

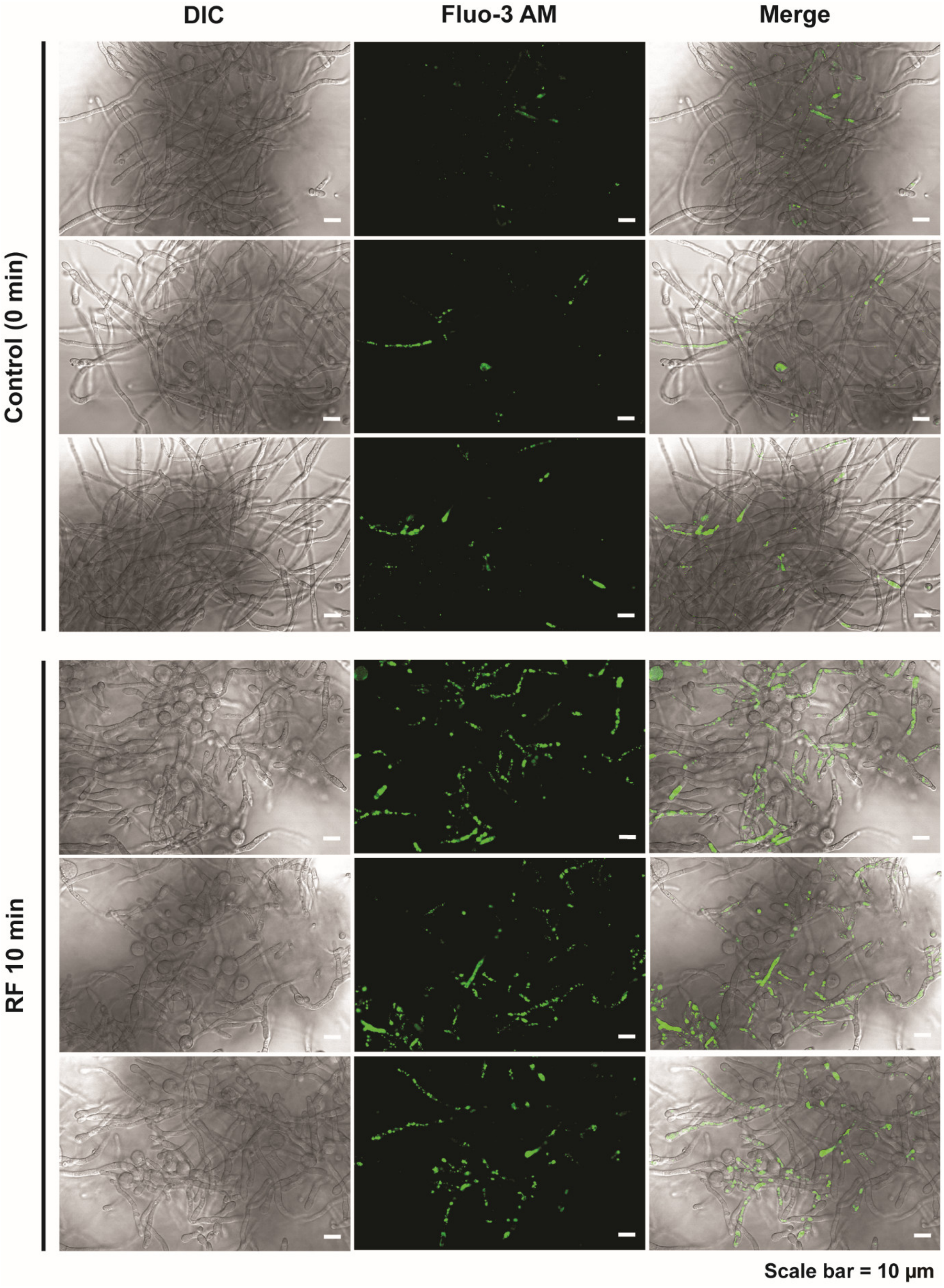
DIC
Fluo-3 AM
Merge
Control (0 min)
RF 10 min
Scale bar = 10 μm

Supplementary Figure S7. Staining intracellular Ca^{2+} in fungal hyphae. A fluorescent dye, Fluo-3 AM, was used for staining fungal hyphae at 16 h after spores were exposed to none (control) and RF-EMF (2 GHz, 0.01 W) for 10 min.

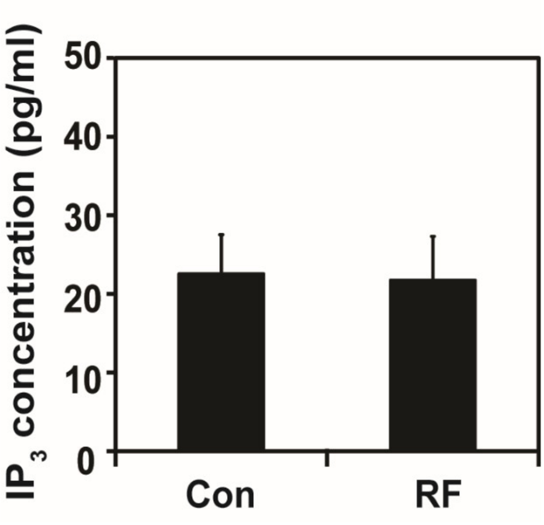

Supplementary Figure S8. Level of IP_3 in fungal hyphae. Fungal hyphae were harvested at 16 h after fungal spores were exposed to none (control) and RF-EMF (2 GHz, 0.01 W) for 10 min. Each value represents the mean of 9 (3 repeated experiment and 3 replicate measurements per each) or 12 (4 repeated experiment and 3 replicate measurements per each) replicate measurements.
